# In Vitro Antileishmanial Activity of Sterols from *Trametes versicolor* (Bres. Rivarden)

**DOI:** 10.3390/molecules21081045

**Published:** 2016-08-10

**Authors:** Vivian Leliebre-Lara, Lianet Monzote Fidalgo, Eva-Maria Pferschy-Wenzig, Olaf Kunert, Clara Nogueiras Lima, Rudolf Bauer

**Affiliations:** 1 Centre for Natural Product Studies, Faculty of Chemistry, University of Havana, Zapata y G, La Habana 10400, Cuba; clara@fq.uh.cu; 2 Parasitology Department, Institute of Tropical Medicine “Pedro Kouri” Marianao 13, Havana 10400, Cuba; monzote@ipk.sld.cu or monzote@hotmail.com; 3 Institute of Pharmaceutical Sciences, Department of Pharmacognosy, University of Graz, Universitaetsplatz 4, 8010 Graz, Austria; eva-maria.wenzig@uni-graz.at (E.-M.P.-W.); rudolf.bauer@uni-graz.at (R.B.); 4 Institute of Pharmaceutical Sciences, Department of Pharmaceutical Chemistry, University of Graz, Universitaetsplatz 4, 8010 Graz, Austria; olaf.kunert@uni-graz.at

**Keywords:** *Trametes versicolor*, trametenolic acid B, antileishmanial activity

## Abstract

Two ergostanes, 5α,8α-epidioxy-22*E*-ergosta-6,22-dien-3β-ol (**1**) and 5α-ergost-7,22-dien-3β-ol (**2**), and a lanostane, 3β-hydroxylanostan-8,24-diene-21-oic acid (trametenolic acid) (**3**), were isolated from an *n*-hexane extract prepared from the fruiting body of *Trametes versicolor* (Bres. Rivarden). The activity of the isolated sterols was evaluated against promastigotes and amastigotes of *Leishmania amazonensis* Lainson and Shaw, 1972. The lanostane, compound (**3**), showed the best inhibitory response (IC_50_ promastigotes 2.9 ± 0.1 μM and IC_50_ amastigotes 1.6 ± 0.1 μM). This effect was 25-fold higher compared with its cytotoxic effect on peritoneal macrophages from BALB/c mice. Therefore, trametenolic acid could be regarded as a promising lead for the synthesis of compounds with antileishmanial activity.

## 1. Introduction

Leishmaniasis is a disease produced by infection with protozoan parasites from the genus *Leishmania* (family Trypanosomatidae). This infection affects humans and numerous species of mammals. The three major clinical leishmaniasis forms occurring in humans, visceral (VL), cutaneous (CL) and mucocutaneous leishmaniasis (MCL), differ in immunopathologies as well as in morbidity and mortality [1]. The number of cases occurring per year is estimated as 0.2–0.4 million for VL, and 0.7–1.2 million for CL, but exact statistical data do not exist [2]. The disease is prevalent in 98 countries worldwide, but 90% of global VL cases occur in only six countries, namely India, Bangladesh, Sudan, South Sudan. Brazil and Ethiopia. CL is more widely distributed, but the majority of cases also occur in poorly developed or developing countries where the access to medical services is usually very limited [1,2].

For treatment of all leishmaniasis forms, pentavalent antimonials have been used as first-line drugs for more than five decades, but high resistance rates have been reported in some regions of India [1,3]. Some alternative treatments include the polyene antifungal amphotericin B, in a liposomal formulation, the alkylphosphocholine miltefosine, and combination therapies [1,4]. Some of these drugs are toxic and produce severe side effects. Moreover, they are expensive and often require long-term use during treatment. Therefore, their availability and affordability in the poorly developed regions where the majority of leishmaniasis cases occur is very restricted [5]. All these problems compelled the search for new antileishmanial agents.

Secondary metabolites from organisms such as higher plants or fungi are a valuable source for the discovery of new bioactive lead structures [6]. Many natural products with the most complex and diverse structures have been isolated from a great variety of organisms and have shown the potential of antileishmanial activity; however, since leishmaniasis is a neglected disease, none of them has undergone clinical evaluation [1,7,8,9,10].

On the other hand, it has to be considered that leishmaniasis is most widespread among the poor population in less developed regions of the world that have only limited access to health services, and that often rely on traditional treatments including native plants and herbal preparations as the only source of primary healthcare. For these people, the use of herbal preparations with proven efficacy constitutes an important therapeutic option, since such preparations are usually more accessible and affordable than conventional synthetic drugs [11,12,13].

The vast structural diversity of natural compounds found in mushrooms provides unique opportunities for discovering new drugs. Experience from ethno-medicine, together with extensive basic laboratory findings, has shown for many years that mushrooms could play an important role in the prevention and treatment of many diseases [14,15,16]. Basidiomycetes, in particular polypores, have a long history of medicinal use [17]. *Trametes versicolor* (Bres. Rivarden) (Polyporaceae) is probably one of the most appreciated non-edible mushrooms as it is the source of the well-studied antitumor polysaccharide Krestin (PSK). PSK, isolated from *T. versicolor*, is a protein-bound polysaccharide or glycoprotein that is effective in several animal models of cancer [18,19]. The other big group of metabolites isolated from this mushroom are lipids, including hydrocarbons, fatty acids, triterpenoids and sterol derivatives [20,21].

Previously, we studied the in vitro antileishmanial activity of different extracts from *T. versicolor*, and the *n*-hexane extract turned out to be moderately active [22]. In the present work, we report the phytochemical investigation of this extract that resulted in the isolation of three triterpenes: two ergostane types and a lanostanoid derivative. The leishmanicidal activity of these triterpenes was evaluated against promastigotes and intracellular amastigotes of *Leishmania amazonensis* (Lainson and Shaw).

## 2. Results

### 2.1. Compound Isolation

**Figure 1:**
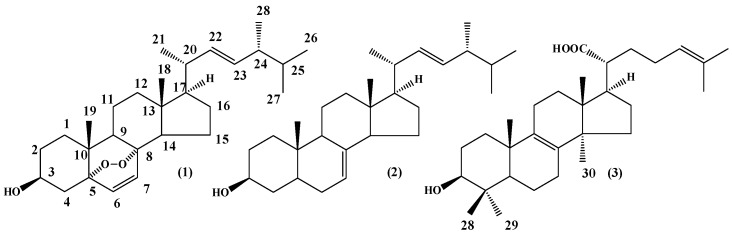
Chemical structure of sterols isolated from *Trametes versicolor* fruiting body. Ergosterol peroxide **1**; 7,22-ergostadien-3β-ol **2** and trametenolic acid B **3**.

*Trametes versicolor* fruiting bodies were extracted by maceration over a week with *n*-hexane to afford 0.42 g dry extract (yield 7.8%) This extract was fractionated by a series of silica gel column chromatographic separation steps. The first fractionation step resulted in 17 combined fractions (A–Q). Fractions A–C had an oily appearance, suggesting the presence of fatty acids and fatty acid esters. From further fractionation, three known sterols were isolated and identified. The structures of the three compounds are presented in Figure 1. On the basis of comparison of spectral ^1^H-NMR, ^13^C-NMR and physical data with literature values, fraction P was identified as 5α,8α-epidioxy-22*E*-ergosta-6,22-dien-3β-ol (ergosterol peroxide) **1** [23], fraction N was found to be 5α-ergost-7,22-dien-3β-ol **2** [24] and the compound in fraction EF was identified as a lanostane-type 3β-hydroxylanostan-8,24-diene-21-oic acid (trametenolic acid B) **3** [25].

The remaining fractions were found to contain a mixture of the isolated compounds in varying proportion as major constituents, together with minor concentrations of other compounds that could not be isolated in sufficient amounts to allow their identification.

### 2.2. Leishmanicidal Activity

The activities of the three sterols against promastigotes and intracellular amastigote forms of *L. amazonensis*, as well as their cytotoxicity on mouse peritoneal macrophages, were tested. The results are summarized in Table 1.

After 72 h of exposure against *L. amazonensis* promastigotes, compounds **1** and **3** inhibited parasite growth at the concentrations of 13.9 ± 0.2 µM and 2.9 ± 0.1 µM, respectively. The ergostane **2** was found to be inactive for the extracellular and the intracellular stages with IC_50_ values exceeding 50 µM for the former and 12 µM for the latter. Compounds **1** and **3** also showed inhibitory effects against the clinically relevant intracellular amastigote form of the parasite with an inhibitory concentration of 4.0 ± 0.1 µM and 1.6 ± 0.1 µM, respectively. However, in both used models, trametenolic acid B **3** produced the most potent antileishmanial activity compared with other isolated compounds. Besides, the three isolated compounds have similar toxicity values ranging from 37.5 ± 6.2 µM to 42.9 ± 2.2 µM, and compound **3** was 25 times more selective. So trametenolic acid B **3** was statistically even more active (*p* < 0.05) than the positive control pentamidine.

## 3. Discussion

Ergosterol peroxide **1** has been isolated from a variety of fungi, yeasts, lichens and sponges [26,27], and has been reported to exhibit immunosuppressive [28], antiviral [29], anti-inflammatory, antitumor [30,31], and trypanocidal [32] activities in vitro. Then 5α-Ergost-7,22-dien-3β-ol **2** was isolated from *Ganoderma applanatum* (Pers.) Pat, among other mushrooms, and proved to be weakly active against a number of Gram-positive and Gram-negative micro-organisms [33]. To the best of our knowledge, there are no previous reports about trametenolic acid B **3** isolation from *T. versicolor* mushrooms, although the compound has been found in many other mushrooms and has been shown to possess cytotoxic and antimicrobial activities [34,35,36].

For testing in vitro antileishmanial activity of the isolated constituents, we used promastigote and amastigote cultures of *L. amazonensis*, a parasite responsible for cutaneous leishmaniasis in regions of the New World. Lately, it has become a focus of the scientific community’s attention as its dissemination can produce a wide spectrum of diseases including mucosal, visceral and post kala-azar dermal leishmaniasis and an atypical American visceral leishmaniasis with hepatitis and lymphadenopathy [37]. Therefore, *L. amazonensis* was the selected species for this study. *Leishmania* screening is usually done on promastigote cultures, because this assay is easy, reproducible and quick; however, promastigotes are not the infective form in vertebrate hosts. So, this preliminary evaluation must be accompanied by the evaluation of intracellular amastigotes in macrophages, which is the clinically relevant stage of *Leishmania* in mammalian hosts. The compound’s cytotoxicity was assayed using non-parasitized macrophages; this value was compared with the activity on amastigotes to evaluate whether the positive in vitro response of the compounds was due to their cytotoxicity or to a selective activity against *Leishmania* [38].

Among the isolated compounds, compound **3** (trametenolic acid B) showed the best in vitro antileishmanial activity and selectivity, which are important criterions to select a new leishmanicidal drug [38]. Therefore, compound **3** could be considered for further study for in vivo antileishmanial activity on one hand, and to elucidate its mechanism of action on the other hand.

While compound **2** was inactive, compound **1** showed moderate leishmanicidal activity in our model. This compound has been reported to exhibit leishmanicidal activity against intracellular amastigotes in a slightly different in vitro model [39]. The mechanism of action proposed for this compound involves the replacement of ergosterol, a structural component of the parasite cell membrane, by ergosterol peroxide during the replication process. The subsequent breaking of the peroxide bond triggers a series of free radical reactions, which may cause the disruption of the parasite membrane [32].

Currently, the molecular mechanism for the antileishmanial activity of the identified compounds has not been studied in detail. Nevertheless, it is well known that sterol biosynthesis is a vital pathway in various species of *Leishmania* parasites, as well as in fungi. It is involved in cell growth and other vital functions [40]. The major sterols found in the promastigote stage of this parasite are ergosterol-related structures with a similarity to compound **2**. Therefore, accumulations of this toxic sterol during treatment do not significantly alter the parasite’s subsistence [41]. In contrast, no lanostane-type compounds have been found during sterol biosynthesis in *L. amazonensis* promastigotes, although they are produced after some medical treatments [42]. Consequently, compound **3** as a lanostane type might cause perturbations in the parasite’s sterol composition and biosynthesis. Also, in the amastigote stage, compound **3** might produce modifications, suggesting that changes in sterol composition are linked to the disturbance of cell membrane structure and function, and hence to parasite death.

Although the *n*-hexane crude extract only showed moderate activity in a preliminary in vitro leishmanicidal assay [22], trametenolic acid B **3** as one of its constituents showed a strong inhibitory activity. This could be due to the low concentration of the sterol in the crude *n*-hexane extract or to an antagonism among constituents.

## 4. Materials and Methods

### 4.1. Mushroom Material

*Trametes versicolor* fruiting bodies were collected from a dead trunk, at Turquinos’s National Park, Santiago de Cuba. A voucher specimen (No. 9805) was deposited at the Herbarium of the National Botanical Garden in Havana, Cuba. The taxonomic identification of the mushroom was performed by the Mycology Department at the Systematic and Ecology Institute in Havana, Cuba, according to the method described by Decock and Herrera [43]. Mushroom material was collected following recommendation by Stamets and Chilton [44].

### 4.2. Extract Preparation and Metabolites Isolation

The mushroom fruiting bodies (250 g) were dried, milled, and subsequently subjected to a solid-liquid extraction with *n*-hexane for a week, at room temperature. The extraction steps yielded 0.42 g of an oily *n*-hexane extract (*n*-hE). The organic solvents were removed under reduced pressure at 35 ± 2 °C using a rotary evaporator (Büchi, Flawil, Switzerland). The *n*-hE was subjected to silica gel column chromatography with an *n*-hexane/EtOAc gradient (9:1–3:7, *v*/*v*). Thin layer chromatography (TLC, silica gel 60 TLC aluminum sheets 20 cm × 20 cm, F254, Merck, Darmstadt, Germany) was used to analyze the collected fractions. Developed plates were sprayed with anisaldehyde/H_2_SO_4_ reagent and then heated at 105 °C. The collected fractions were combined based on their TLC profile to yield 17 combined fractions (Fraction A to Q). The P and N fractions were separately re-crystallized in methanol and *n*-hexane to provide 13.2 mg of compound **1** and 8.7 mg of compound **2** respectively. The E and F fractions were combined and submitted to silica gel column chromatography, eluting with a solvent mixture of *n*-hexane/EtOAc (4:1–1:1, *v*/*v*), to yield six fractions. Fraction EF-5 was re-crystallized in methanol to furnish 8.4 mg of compound **3** (Figure 1).

### 4.3. Structure Elucidation

The 1D and 2D NMR spectra for compounds **1** and **2** were recorded by a Varian VNMRS 600 NMR spectrometer (Varian, Palo Alto, CA, USA) operating at a proton NMR frequency of 599.83 MHz using a 5 mm inverse detection cryoprobe. ^1^H-NMR spectra were recorded with digital resolution 0.367 Hz/point. The 1D and 2D NMR spectra were recorded at 600 MHz (^1^H) and 125 MHz (^13^C) on a Varian^®^ (Varian) NMR spectrometer using TMS as the internal standard with CDCl_3_ as solvent for the compound **3**. ESI-MS spectra were recorded on an LTQ-XL ion trap mass spectrometer (Thermo Fisher Scientific, Waltham, MA, USA) in the ESI positive mode (capillary temperature: 330 °C; source voltage: 4 kV; capillary voltage: 35 V). EI mass spectra were recorded on an Agilent HP7890A GC coupled to a 5975C VL MSD (Agilent Technologies, Santa Clara, CA, USA). GC separations were performed on a HP5-MS capillary column (ID 0.25 mm, length 30 m, film thickness 0.5 µm; Agilent Technologies). Helium was used as carrier gas (0.9 mL/min). Injector temperature was 240 °C and injector temperature 280 °C.

Compound **1** (Ergosterol-5α,8α-peroxide): ^1^H-NMR (400 MHz, CDCl_3_): (δ, ppm) 0.82 (s, CH_3_-18), 0.82 (d, *J* = 6.6 Hz, CH_3_-26), 0.84 (d, *J* = 6.6 Hz, CH_3_-27), 0.89 (s, H-19), 0.92 (d, *J* = 7.0 Hz, CH_3_-28), 1.05 (d, *J* = 6.6 Hz, CH_3_-21), 3.97 (m, CH-3), 5.15 (dd, *J*_1_ = 15.0 Hz, *J*_2_ = 8.4 Hz, CH-23), 5.20 (dd, *J*_1_ = 15.0 Hz, *J*_2_ = 7.8 Hz, CH-22), 6.55 (d, *J* = 8.4 Hz, CH-7), 6.26 (d, *J* = 8.4 Hz, CH-6); ^13^C-NMR (δ, ppm): 12.8 (C-18), 17.5 (C-28), 19.6 (C-19), 19.6 (C-26), 19.9 (C-27), 23.4 (C-15), 20.9 (C-21), 20.6 (C-11), 28.6 (C-16), 30.1 (C-2), 33.0 (C-25), 34.7 (C-1), 36.9 (C-4), 36.9 (C-10), 39.3 (C-12), 39.7 (C-20), 42.7 (C-24), 44.5 (C-13), 51.1 (C-9), 51.6 (C-14), 56.2 (C-17), 66.5 (C-3), 82.1 (C-8), 79.4 (C-5), 130.7 (C-7), 132.3 (C-23), 135.2 (C-22), 135.4 (C-6).

Compound **2** (ergosta-7,22-dien-3β-ol): ^1^H-NMR (400 MHz, CDCl_3_): (δ, ppm) 0.82 (s, CH_3_-18), 0.82 (d, *J* = 6.6 Hz, CH_3_-26), 0.84 (d, *J* = 6.6 Hz, CH_3_-27), 0.89 (s, H-19), 0.92 (d, 7.0 Hz, CH_3_-28), 1.07 (d, *J* = 6.6 Hz, CH_3_-21), 3.6 (m, CH-3), 5.19 (t, *J* = 6.9 Hz, CH-7), 5.15 (m, 2H, CH-22, CH-23), ^13^C-NMR (δ, ppm): 37.9 (C-1), 31.5 (C-2), 71.6 (C-3), 37.1 (C-4), 49.5 (C-5), 29.7 (C-6), 117.4 (C-7), 139.5 (C-8), 40.5 (C-9), 34.2 (C-10), 21.5 (C-11), 39.4 (C-12), 43.3 (C-13), 50.1 (C-14), 22.9 (C-15), 28.6 (C-16), 55.9 (C-17), 13.0 (C-18), 17.6 (C-19), 40.2 (C-20), 21.01(C-21), 131.8 (C-22), 135.7 (C-23), 42.8 (C-24), 33.1 (C-25), 19.6 (C-26), 19.9 (C-27), 17.6 (C-28). EI-MS: *m*/*z* 398.

Compound **3** (Trametenolic acid B): ^1^H-NMR (600 MHz, Py-*d*_5_): (δ, ppm) 1.09 (s, CH_3_-18),1.26 (s, CH_3_-28), 1.02 (s, CH_3_-19), 1.03 (s, CH_3_-30), 1.08 (s, CH_3_-29), 1.68 (s, CH_3_-26), 1.63 (s, CH_3_-27), 2.65 (dd, *J*_1_ = 11.1, *J*_2_ = 8.2 Hz, H-20), 3.45–3.40 (m, H-3α), 5.32 (t, *J* = 7.1 Hz, H- 24). ^13^C-NMR (δ, ppm): 36.5 (C-1), 29.1 (C-2), 78.4 (C-3), 39.9 (C-4), 51.3 (C-5), 19.1 (C-6), 27.2 (C-7), 134.7 (C-8), 135.5 (C-9), 37.8 (C-10), 21.6 (C-11), 29.8 (C-12), 45.3 (C-13), 50.2 (C-14), 31.2 (C-15), 27.9 (C-16), 49.4 (C-17), 16.8 (C-18), 19.8 (C-19), 48.1 (C-20), 179.0 (C-21), 33.7 (C-22), 27.1 (C-23), 125.3 (C-24), 132.1 (C-25), 26.1 (C-26), 18.1 (C-27), 28.9 (C-28), 16.7 (C-29), 24.9 (C-30). ESI-MS+: *m*/*z* 457.3 [M + H]^+^; 439.4 [M + H − H_2_O]^+^ 421.4: [M + H − 2H_2_O]^+^.

### 4.4. Parasites

Strain MHOM/77BR/LTB0016 of *L. amazonensis* was kindly provided by the Department of Immunology, Oswaldo Cruz Foundation (FIOCRUZ), Brazil. Parasites were routinely isolated from lesions in BALB/c mice as amastigotes and transformed into promastigotes at 28 °C in Schneider’s medium (Sigma-Aldrich, St. Louis, MO, USA) containing 10% heat-inactivated fetal bovine serum (HFBS) (Sigma-Aldrich) and respective antibiotics (100 μg of streptomycin/mL plus 100 U of penicillin/mL). Parasites were maintained as promastigotes with passages every three or four days and only were used before 10 in vitro passages.

### 4.5. Anti-Promastigote Assay

Activity testing against promastigotes was carried out in 96-well plates. Exponentially growing cells (10^5^ promastigotes/mL, 199 μL) were distributed in 96-well plates. One microliter of compounds in stock solution at 2 mg/mL or 1 μL of dimethylsulfoxide (DMSO) for control was added at different concentrations between 1.25, 2.5, 5.0, 10.0 and 20 µg/mL, and incubated at 28 °C for 72 h. Then, the parasites were incubated for additional 4 h with 20 µL of 3-[4,5-dimethylthiazol-2-yl]-2,5-diphenyltetrazolium bromide (MTT) (Sigma-Aldrich), prepared at 5 mg/mL and filtered just prior to use. After incubation, the formazan crystals were dissolved by addition of 100 μL of DMSO. The optical density was determined using a spectrophotometer (Sirio S Reader, 2.4-0, Florence, Italy), at a test wavelength of 560 nm and a reference wavelength of 630 nm [45]. Inhibitory activity (%) of each concentration respect to control cultures was calculated.

### 4.6. Anti-Amastigote Activity

The activity against intracellular amastigotes was evaluated by the method described by Caio et al. [46]. Peritoneal macrophages from normal BALB/c mice were harvested at moment of use in cold RPMI medium (Sigma-Aldrich) and antibiotics. Macrophages were plated in 24-Well Lab-Tek (Costar^®^, New York, NY, USA) at 5 × 10^5^ cells/mL and the plate was incubated at 37 °C under an atmosphere of 5% CO_2_ for 2 h. Non-adherent cells were removed by washing with phosphate buffer. Then, *L. amazonensis* promastigotes in stationary phase were added at a 4:1 parasite/macrophage ratio and the cultures were subsequently incubated for further 4 h at same conditions. Free parasites were also removed by washing and 1995 μL of RPMI medium (Sigma-Aldrich) containing 10% HFBS and antibiotics were added in the first well. Additionally, 5 μL of the different compounds concentrations (0.3, 0.6, 1.25, 2.5 and 5.0 μg/mL) dissolved in DMSO were added in duplicate and the plate was incubated for further 48 h at 5% CO_2_ and 37 °C. Finally, supernatant was eliminated by washing cultures, were fixed in absolute methanol, stained with Giemsa, and examined under immersion oil in a light microscope at 1000×. The number of intracellular amastigotes was determined by counting them in 100 macrophages per sample and calculating the percentage of infected macrophages. The results were expressed as percent of infection reduction rate in comparison with that of the controls treated with DMSO, where the infection rates were obtained by multiplying the percentage of infected macrophages by the number of amastigotes per infected macrophages.

### 4.7. Cytotoxicity Assay

Peritoneal macrophages were collected as described previously seeded at 5 × 10^5^ cells/mL in a 96-well plate and incubated for 2 h at 37 °C in 5% of CO_2_. Free cells were removed and dilutions of compounds in 1 μL of DMSO were added in 199 μL of RPMI medium with 10% HFBS and antibiotics. The macrophages were treated with six concentrations of each compound (1.25, 2.5, 5.0, 10.0, 20.0 and 40.0 μg/mL). Cultures treated with 1 μL of DMSO were included as controls. Cytotoxicity was determined after 72 h of incubation using the colorimetric assay with MTT as previously described, although in this case, 15 μL of MTT were added to each well. After incubation for 4 h, formazan crystals were dissolved with 100 μL of DMSO, and optical density was determined at 560 nm, using a reference wavelength of 630 nm [47]. Then inhibitory activity (%) of each concentration respect to control cultures was determined.

### 4.8. Statistical Analyses

The medium inhibitory concentration (IC_50_) to parasite assay and medium cytotoxic concentration (CC_50_) was obtained from linear dose-response. Results are expressed as mean ± standard deviation of three independent replicates. Selectivity indexes (SI) were calculated by dividing the CC_50_ for peritoneal macrophages of BALB/c mice by the IC_50_ for *L. amazonensis* amastigotes [48]. Statistical differences, classified as *p* < 0.05, between IC_50_ of compounds were determined via Mann-Whitney test using the STATISTICA for Windows Program (Release 4.5, StatSoft, Inc., Tulsa, OK, USA).

## 5. Conclusions

Three sterols, ergosterol peroxide **1**, ergost-7, 22-dien-3-ol **2** and trametenolic acid B **3**, were isolated from *n*-hexane extract from the fruiting body of *T. versicolor*. Trametenolic acid B **3** is reported for the first time in this mushroom species. The three pure compounds showed in vitro effects against *L. amazonensis*, and particularly compound **3** was the most active and highly selective against both intracellular and extracellular stages of *L. amazonensis.* These findings corroborate the antileishmanial potential of *T. versicolor* and make this mushroom, which grows wildly in many tropical countries where a high number of leishmaniasis cases occur, an interesting option for phytotherapeutic leishmaniasis treatment that should be further studied in more detail. The most active constituent, trametenolic acid B **3** might constitute an interesting “hit” with potential leishmanicidal activity. Further studies will be necessary to verify the activity of this compound in animal models and to elucidate its mechanism of action. Since a potential target of the identified active compounds is the parasitic sterol biosynthesis and/or cell wall incorporation, further studies with structurally related ergostane and lanostane-type triterpenes are considered very useful to get a better idea of the leishmanicidal potential of this class of compounds.

## Figures and Tables

**Table 1:** Antileishmanial and cytotoxic activity of isolated compounds from *Trametes versicolor*.

Compounds	IC_50_^a^ ± SD ^b^				CC_50_^c^ ± SD ^b^		SI ^d^
	Promastigotes		Amastigotes		Macrophages		
	µg/mL	µM	µg/mL	µM	µg/mL	µM	
**1**	5.9 ± 0.1	13.9 ± 0.2	1.7 ± 0.1	4.0 ± 0.1	18.4 ± 0.9	42.9 ± 2.2	11
**2**	>20	>50	>5	>12	14.9 ± 4.9	37.5 ± 6.2	-
**3**	1.3 ± 0.03	2.9 ± 0.1	0.7 ± 0.03 *	1.6 ± 0.1 *	18.0 ± 2.8	39.4 ± 4.8	25
**Pentamidine ^e^**	0.4 ± 0.01	1.2 ± 0.02	1.3 ± 0.1	3.8 ± 0.2	11.7 ± 1.7	34.4 ± 4.9	9

^a^ IC_50_: Medium inhibitory concentration. Concentration that causes 50% of growth inhibition; ^b^ SD: Standard deviation of three replicates; ^c^ CC_50_: Medium cytotoxic concentration. Concentration that causes 50% of mortality; ^d^ SI: Selectivity index. CC_50_ for macrophages (µM)/IC_50_ for intracellular amastigotes (µM); ^e^ Pentamidine: Reference drug; * Significant higher activity (*p* < 0.05) compared with pentamidine.
